# Zenithal alignment of liquid crystal on homeotropic polyimide film irradiated by ion beam

**DOI:** 10.1186/1556-276X-7-63

**Published:** 2012-01-05

**Authors:** Yoonseuk Choi, Tae-Hoon Yoon, Jin Hyuk Kwon, Jonghoon Yi, Jin Seog Gwag

**Affiliations:** 1 Department of Electronics, Hanbat National University, Daejeon, 305-719, South Korea; 2 Department of Electronics Engineering, Pusan National University, Pusan, 609-735, South Korea; 3 Department of Physics, Yeungnam University, 214-1 Dae-dong, Gyeongsan 712-749, South Korea

## Abstract

We investigate the pretilt characteristics of a nematic liquid crystal [LC] in terms of ion beam exposure conditions on the homeotropic polyimide alignment layer. The pretilt angle of LCs in the case of high-energy ion beam treatment was decreased considerably almost the same to that of the homogenous alignment layer though we used homeotropic polyimide film at first. Increasing irradiating energy, we could control the pretilt from 90° to 1° with several steps. We believe that this is because the side chain with hydrophobicity in the used polyimide is broken by ion beam exposure. To confirm it, contact angle measurement was carried out. With this result, we can easily control the LC pretilt in the pixel with appropriate exposure conditions which is critical to achieve excellent electrooptic characteristics and good image quality.

## Introduction

In practical applications of liquid crystals [LCs], the study of the LC alignment near the surface including in-plane and out-of-plane directions is crucial. Various alignment techniques have been introduced to create physical/chemical anisotropy on the surface of the alignment layer [1-11]. The most representative skill used in industry is the rubbing method which has high productivity. However, since rubbing is a contact method, it often produces a fine scratch on the alignment layer during the process, and it results in several disadvantages, such as the rubbing mura at a dark level of liquid crystal displays [LCDs].

To overcome such problems, various alternative non-contact methods are suggested for high-resolution LCDs such as photo-alignment technique with ultraviolet [UV] light and ion beam technique [1,7]. The polarized UV irradiation technique has many merits such as a clear image without the mura at a black level and an easy creation of multi-domain in a pixel. However, it also has a problem such as an image-sticking issue due to a low-surface anchoring strength.

Since the extremely collimated ion beam to confirm a high linear motion can induce an excellent anisotropy on the polyimide surface by selective destruction of π-bonding which plays an important role in the alignment of LCs, it could be a good option of non-contact surface modification method [2,10]. Also, this method is relatively free of the image-sticking problem since it generates a strong anchoring strength like the rubbing method. Therefore, we can conclude that an appropriate LC pretilt control technique considering LCD modes is essential to obtain better electrooptical characteristics of LCDs and to develop new LC device applications.

In this paper, we investigate the pretilt characteristics of a nematic LC in terms of ion beam irradiation conditions using homeotropic polyimide alignment layer. The modification of LC pretilt with different ion beam exposure parameters was studied experimentally. As a result, we successfully changed the pretilt angle from 90° to 1° with several sub-steps.

### Experimental details

**Figure 1:**
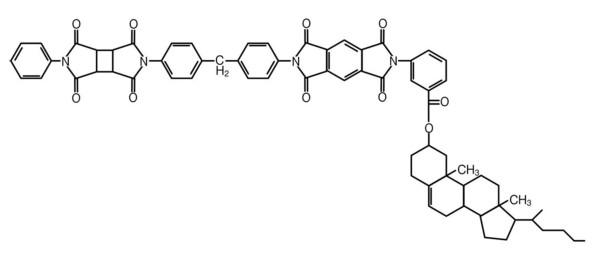
**The chemical structure of the polyimide AL-00010 used as the LC alignment layer**.

The glass substrates coated with indium tin oxide whose size is 30 mm × 20 mm × 0.7 mm are spin-coated with the polyimide AL-00010, which is provided by Japan Synthetic Rubber Corporation (Minato-ku, Tokyo, Japan). It subsequently was prebaked at 80°C for 3 min to remove the solvent and cured at 230°C for 30 min for polymerization on a hot plate. Figure 1 shows the chemical structure of the polyimide AL-00010. In general, the pretilt angle of LCs is determined mainly by the chemical property of the side chain in the polyimide. Since the side chain in the AL-00010 has hydrophobicity with a nonpolar molecule, we can easily know that it may align LCs vertically.

After coating and baking, this homeotropic polyimide film was bombarded by an argon ion beam to change the LC aligning properties of the surface. In this experiment, various ion beam exposure conditions such as the irradiation energy, exposure angle, exposure time, and current density are modulated to examine how the surface property is changed according to them. As an ion source, a cold hollow cathode [CHC] is used to yield a highly collimated ion beam. In order to collimate the ion beam, two perforated grids are used as electro-focusing lenses. The CHC represents a separate cooled chamber which is equipped with a magnetic system and is connected to a discharge chamber through an orifice. Argon gas feeding into the ion source is carried out through the CHC only.

In order to investigate the pretilt angle of treated polyimide surfaces, we had fabricated several LC cells by using the substrates with different ion beam exposure conditions. The cell was filled with a nematic LC (MLC-6610 from Merck KGaA, Darmstadt, Germany) with negative dielectric anisotropy, and the cell gap was maintained by 12-μm glass spacers. The LC injection was carried out at room temperature. The pretilt angle was measured by the extended crystal rotation method which can measure a wider range of the pretilt [12,13].

## Results and discussion

**Figure 2:**
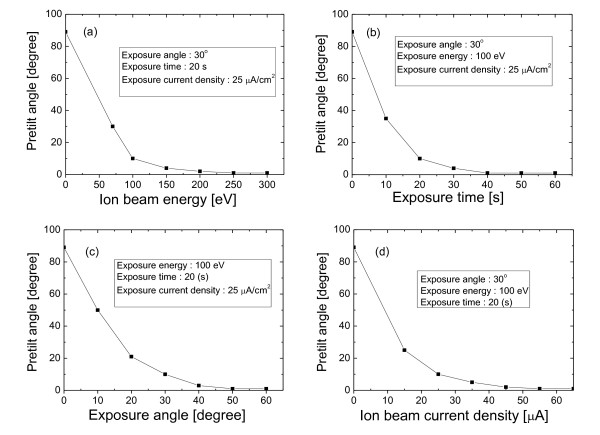
**LC pretilt angle depending on ion beam exposure conditions**. (**a**) LC pretilt angle as the function of ion beam energy, (**b**) LC pretilt angle as the function of exposure time, (**c**) LC pretilt angle as the function of exposure angle, and (**d**) LC pretilt angle as the function of ion beam current density.

As shown in Figure 2, we can see that the pretilt angle of the LC is aligned almost horizontally in some ion beam conditions such that the ion beam energy is higher than 150 eV when exposure angle, exposure time, and ion beam current density are 30°, 20 s, and 25 μA, respectively. This may indicate that the side chain with hydrophobicity in the polyimide AL-00010 is destroyed or broken off by ion beam particles, and eventually, the average polarity of the used surface has changed. In general, the remaining portion of the side chain on the polyimide surface determines the LC pretilt on average.

To confirm our speculation about the change of the surface property of AL-00010 after ion beam exposure, we had measured the surface contact angle. The contact angle is described as the angle between the solid surface and the tangent line of liquid at the solid-liquid-vapor interface when the liquid is in a thermal equilibrium state on the solid. The equilibrium state of the contact angle which represents minimum total interfacial energy is obtained when the vector sum of the horizontal force components among the solid-liquid, liquid-vapor, and solid-vapor interface is zero. This method has a merit which can measure the surface property without any surface damage. Generally, the wetting of liquid at the interface is a result to reduce the interfacial energy. The spreading parameter *S *related to interfacial tension can be defined by

(1) $$S=\gamma_{\text{sv}}-\gamma_{\text{sl}}-\gamma_{\text{lv}}$$

Here, *γ*_SV _is tension between solid and vapor, *γ*_Sl _is tension between solid and liquid, and *γ*_lV _is tension between liquid and vapor. In Equation 1, when *S *≥ 0(*γ*_Sl _+ *γ*_lV _≤ *γ*_SV_), the solid is wetted by the liquid entirely, and then, it is called complete wetting. If *S *< 0(*γ*_Sl _+ *γ*_lV _≤ *γ*_SV_), then the solid prefers the vapor to the liquid; however, since the liquid is heavier than the vapor, the liquid is placed under the vapor, and then, the solid is wetted partially by the liquid. Thus, it is called partial wetting. Young's equation describing the relation of the three interfacial tensions in equilibrium state is written as

(2) $$\gamma_{\text{lv}}\cos\theta =\gamma_{\text{sv}}-\gamma_{\text{sl,}}$$

where *θ *is the contact angle. As known in Equation 2, when the liquid is water with chemical polarity, the lower the contact angle is, the higher the surface energy and the hydrophilic property are. Meanwhile, the higher the contact angle is, the lower the surface energy and the hydrophilic property are.

In this measurement, we used distilled water having chemical polarity as a liquid. The ion beam-treated polyimide AL-00010 substrate of which the ion beam conditions are ion beam energy of 300 eV, ion beam current density of 50 μA/cm^2^, exposure angle of 30°, and exposure time of 30 s was used as a sample. The ion beam condition is enough to modify the surface as shown in Figure 2. As a comparison, the sample with untreated polyimide AL-00010 substrate was examined.

**Figure 3:**
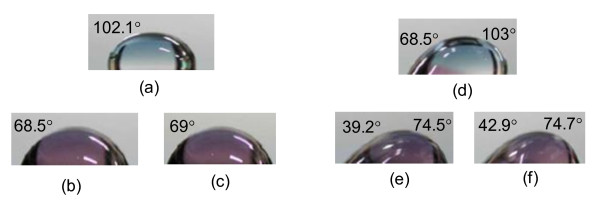
**The contact angle of droplet on a sample before and after ion beam treatment**. For static mode, (**a**) the droplet image on the sample before ion beam treatment, (**b**) the droplet image taken parallel to the ion beam-irradiated direction after ion beam treatment, and (**c**) the droplet image taken perpendicular to the ion beam-irradiated direction after ion beam treatment. For dynamic mode, (**d**), (**e**), and (**f**) show that the angle difference is smaller for the direction perpendicular to the ion beam exposure.

The contact angle at static mode was reduced seriously after ion beam treatment as shown Figure 3a, b, c. Figure 3a is a droplet image taken on a sample before ion beam treatment. On the other hand, Figure 3b, c shows droplet images taken parallel and perpendicular to the ion beam-irradiated direction for the ion beam-treated sample, respectively. This result supports obviously the analysis that the side chain with hydrophobicity in the polyimide broke off or was destroyed by the ion beam as expected in the sharp decrease of the LC pretilt (see Figure 2). In an additional experiment, we measured the dynamic contact angle of this sample. The dynamic contact angle is the contact angle before slipping down the liquid on the substrate when we tilt the sample slowly and informs us macroscopically of the relative information about the surface roughness. Generally, if the angle difference between the advancing angle and receding angle is larger, then the surface is rougher. Figure 3d, e, f shows that the angle difference is small for the direction perpendicular to the ion beam exposure. Figure 3d is taken before treatment, and Figure 3e, f images are taken in the case of parallel and perpendicular ion beam irradiation, respectively. This means that the roughness of the surface in the case of parallel irradiation is bigger than that that of the perpendicular irradiation case. Note that this matches well to other ion beam treatment results [14]. Consequently, we verify what the LC pretilt change causes in ion beam exposure from the contact angle method.

**Figure 4:**
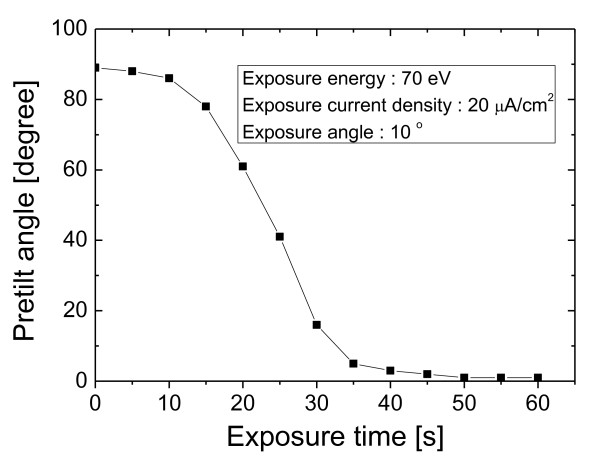
**LC pretilt angle according to exposure time at optimized ion beam parameters**.

Based on the relationship between the ion beam exposure condition and the pretilt angle indicated in Figure 2, to enlarge the margin of LC pretilt control by ion beam irradiation, the ion beam conditions for our ion beam equipment were optimized and were 70 eV, 20 μA/cm^2^, and 10°. In our ion beam equipment, ion beam irradiation with an ion beam energy under 70 eV generated a large fluctuation of beam current. Figure 4 shows the change of the LC pretilt according to exposure time at the optimized condition. As you see, we can know that the LC pretilt margin is enlarged extensively compared with Figure 2. We would get a larger margin of the LC pretilt if we could have a better one.

## Conclusion

In summary, we investigated the LC pretilt property according to the ion beam exposure conditions on a homeotropic LC alignment. The LCs on a higher energy ion beam-treated surface is aligned almost homogenously. We assumed that it may be caused by the breaking of the side chain with hydrophobic property in the used polyimide by ion beam exposure. It was confirmed from the result of the contact angle measurement. LC pretilt which may create new LCD applications with excellent electrooptic characteristics and good image quality can be controlled by an ion beam condition with proper ion beam parameters.

## Competing interests

The authors declare that they have no competing interests.

## Authors' contributions

YC carried out the sample preparation and measurement and drafted the manuscript. THY and JHK participated in the design of the study and performed the analysis. JY carried out the ion beam exposure and measurement. JSG conceived the study, participated in its design and coordination, and drafted the manuscript. All authors read and approved the final manuscript.
